# A Structurally Optimized and Efficient Lightweight Object Detection Model for Autonomous Driving

**DOI:** 10.3390/s26010054

**Published:** 2025-12-21

**Authors:** Mingjing Li, Junshuai Wang, Shuang Chen, LinLin Liu, KaiJie Li, Zengzhi Zhao, Haijiao Yun

**Affiliations:** 1 College of Electronic Information Engineering, Changchun University, Changchun 130022, China; limj@ccu.edu.cn (M.L.); 240401173@mails.ccu.edu.cn (J.W.); 250402252@mails.ccu.edu.cn (L.L.); 250402232@mails.ccu.edu.cn (K.L.); 250402237@mails.ccu.edu.cn (Z.Z.); yunhj@ccu.edu.cn (H.Y.); 2 School of Information and Electronic Engineering, Shangqiu Institute of Technology, Shangqiu 476000, China

**Keywords:** object detection, lightweight design, YOLOv8, C2f-Faster, EMSConv, autonomous driving

## Abstract

Object detection plays a pivotal role in safety-critical applications, including autonomous driving, intelligent surveillance, and unmanned aerial systems. However, many state-of-the-art detectors remain highly resource-intensive; their large parameter sizes and substantial floating-point operations make it difficult to balance accuracy and efficiency, particularly under constrained computational budgets. To mitigate this accuracy–efficiency trade-off, we propose FE-YOLOv8, a lightweight yet more effective variant of YOLOv8 (You Only Look Once version 8). Specifically, two architectural refinements are introduced: (1) C2f-Faster (Cross-Stage-Partial 2-Conv Faster Block) modules embedded in both the backbone and neck, where PConv (partial convolution) prunes redundant computations without diminishing representational capacity; and (2) an EfficientHead detection head that integrates EMSConv (Efficient Multi-Scale Convolution) to enhance multi-scale feature fusion while simplifying the head design and maintaining low computational complexity. Extensive ablation and comparative experiments on the SODA-10M dataset show that FE-YOLOv8 reduces the parameter count by 31.09% and the computational cost by 43.31% relative to baseline YOLOv8 while achieving comparable or superior mean Average Precision (mAP). Generalization experiments conducted on the BDD100K dataset further validate these improvements, demonstrating that FE-YOLOv8 achieves a favorable balance between accuracy and efficiency within the YOLOv8 family and provides new architectural insights for lightweight object detector design.

## 1. Introduction

Object detection, a cornerstone of computer vision, supports safety-critical applications such as autonomous driving, intelligent surveillance, and unmanned aerial systems. In autonomous driving, vehicles must rapidly and accurately localize pedestrians, vehicles, and traffic signs in highly dynamic environments to ensure driving safety and improve traffic efficiency [1]. In recent years, the YOLO (You Only Look Once) family has emerged as the dominant paradigm for real-time detection due to its end-to-end training mechanism and strong feature extraction capabilities. Notably, YOLOv8 achieves state-of-the-art accuracy and robustness, particularly in small-object detection tasks.

Nevertheless, the backbone and head of YOLOv8 still contain substantial parameter redundancy and computational overhead, which limit their efficiency in practical applications. Conventional lightweight techniques—such as network pruning, quantization, and knowledge distillation—can reduce computational complexity; however, they often result in accuracy degradation or incur additional training costs. General-purpose lightweight architectures—such as MobileNet (Mobile Convolutional Neural Network), ShuffleNet (Channel Shuffle Convolutional Neural Network), and GhostNet (Ghost Feature Convolutional Neural Network)—demonstrate competitive performance in image classification tasks but typically exhibit insufficient accuracy and limited generalization in high-precision detection scenarios, including autonomous driving. Consequently, relying solely on these strategies makes it difficult to simultaneously achieve high detection accuracy and computational efficiency.

To address these limitations, we propose FE-YOLOv8, a lightweight yet accurate detector tailored for autonomous driving applications. Specifically, C2f-Faster modules are integrated into both the backbone and neck to prune redundant computations and enhance gradient propagation. In the detection head, an EfficientHead architecture incorporating EMSConv (Efficient Multi-Scale Convolution) [2] is designed to strengthen multi-scale feature fusion and further reduce computational cost while preserving strong representational capability. The three detection scales adopt structurally identical branches with independent parameters. These architectural refinements collectively accelerate inference and maintain competitive accuracy, achieving an appealing balance between precision and computational efficiency. The proposed method enhances lightweight detection performance and is supported by extensive empirical results.

## 2. Related Work

### 2.1. YOLO Object Detection Algorithm

YOLO epitomizes single-shot, end-to-end object detection. By unifying localization and classification within a single convolutional neural network, it achieves real-time inference with minimal latency [3]. Specifically, the input image is divided into an S×S grid, where each cell directly regresses bounding-box coordinates and class probabilities in a single forward pass, formulating object detection as a pure regression task. Compared with two-stage detectors, this streamlined pipeline offers a strong balance between speed and accuracy, making it well suited for time-critical applications.

Architecturally, the original YOLO drew significant inspiration from GoogLeNet [4] (Inception v1 architecture), employing 24 convolutional layers followed by two fully connected layers. Moreover, 1 × 1 bottleneck convolutions were inserted before selected 3 × 3 kernels to compress feature depth, thereby reducing FLOPs (Floating-Point Operations) while preserving representational capacity. Since its introduction, the YOLO family has undergone continuous evolution, achieving consistent improvements in both accuracy and inference speed, and has become a de facto benchmark for real-time object detection.

Since its inception, the YOLO family has undergone successive architectural refinements, continually improving detection accuracy, feature representation, and inference efficiency. YOLOv5 [5] introduced a CSP (Cross-Stage Partial) backbone, a Focus slicing stem, and an SPPF (Spatial Pyramid Pooling–Fast) module to enrich gradient diversity and accelerate model convergence. YOLOv7 [6] proposed ELAN (Efficient Layer Aggregation Network), which reorganized gradient flow to enhance multi-scale feature fusion without increasing parameter count. YOLOv8 [7] evolved ELAN into the more lightweight C2f (Cross-Stage Partial block with Two-Way Feature Fusion), replaced the anchor-based head with an anchor-free design, and incorporated CIoU (Complete Intersection over Union) and DFL (Distribution Focal Loss) as its loss functions, achieving notable improvements—particularly in small-object detection. YOLOv11 [8] further replaced C2f with a C3k2 (Cross-Stage Partial C3 block with Two-Branch Kernel Aggregation) structure, embedded a C2PSA (Cross-Stage Partial with Pointwise Spatial Attention) module, and redesigned the SPPF block to strengthen spatial.

Despite these advances, the increasing use of sophisticated modules inevitably introduces additional parameters and computational overhead. Given that this study emphasizes high detection accuracy under constrained computational budgets, YOLOv8s is selected as the baseline model because it offers a strong balance among performance, inference speed, and computational cost.

### 2.2. Current Research Status of Lightweight Models

Lightweight architectures have gained increasing attention due to their reduced parameter count and lower computational complexity while maintaining competitive accuracy [9]. Consequently, the design of compact networks has become an active research direction, with new micro-blocks, connectivity patterns, and search-based frameworks continually being proposed to minimize model size with limited accuracy degradation. Unlike accuracy-driven flagship detectors, lightweight models prioritize inference efficiency and computational economy. Current network compression strategies can be broadly categorized into four groups: structured pruning, knowledge distillation, post-training quantization, and hand-crafted or NAS-based lightweight backbones [10].

Model quantization converts network parameters into lower-bit representations, substantially reducing memory usage and accelerating inference because lower-precision operations are generally faster. Sun et al. [11] proposed a Taylor-based ranking strategy that integrates pruning and quantization to reduce computational complexity and storage requirements. Boutros et al. [12] introduced QuantFace, a low-bit-precision model for face recognition. Park et al. [13] employed 8-bit quantization in speech recognition to reduce both latency and memory consumption. For network pruning, Peng et al. [14] proposed a threshold-based algorithm that compressed GoogLeNet parameters by a factor of 16. Xu [15] combined L1-norm-based pruning with iterative pruning guided by weight variation. Xiang et al. [16] significantly reduced the parameter count of fluid neural networks using structured pruning with ADMM (Alternating Direction Method of Multipliers) constraints. Regarding knowledge distillation, Blakeney et al. [17] developed a parallel segmented distillation method to accelerate the training of deep neural networks. Kang et al. [18] proposed a data-free distillation approach and extended it to regression tasks. Tung et al. [19] introduced a similarity-preserving distillation technique, enabling student models to learn the relational structure of teacher representations.

Another mainstream direction in model lightweighting is the design of dedicated efficient modules. The MobileNet series [20,21,22] employs depthwise separable convolutions, inverted residual blocks, and the H-Swish activation function to substantially reduce computational cost while maintaining accuracy. The ShuffleNet series [23,24] leverages grouped convolutions and channel-shuffle operations to improve computational efficiency. GhostNet [25] introduces the GhostModule, which generates additional feature maps through inexpensive operations to further enhance efficiency. Although these architectures achieve strong performance on lightweight classification tasks, they still exhibit limitations in high-precision detection scenarios such as autonomous driving. For example, MobileNetV2_CA [26] and attentive-aggregation-based networks [27] have been explored as lightweight detectors, but their performance remains insufficient for accuracy-demanding detection tasks.

In recent years, numerous YOLO-based lightweight detectors tailored for autonomous-driving perception have been proposed. Yang and Fan introduced YOLOv8-Lite, a streamlined variant of YOLOv8 featuring a compact backbone–neck design and a simplified detection head [28]. Cui et al. proposed DAN-YOLO, which incorporates a dilated aggregation network to strengthen long-range contextual modeling with minimal parameter increase, thereby improving the detection of small and distant road objects [29]. Li et al. presented MST-YOLO, a Transformer-enhanced lightweight variant that employs multi-scale attention and feature aggregation to improve small-object detection in autonomous-driving scenes [30]. Kalgaonkar and El-Sharkawy further explored attentive feature aggregation in a lightweight CNN–YOLO hybrid architecture to enhance feature expressiveness for automotive detection tasks under limited computational budgets [31]. In addition, several works incorporate lightweight convolutional modules or MobileNet-derived components into YOLO frameworks, demonstrating that compact convolutional blocks, multi-scale aggregation, and attention-guided refinement can effectively enhance detection robustness in autonomous-driving environments with modest computational cost. Overall, these lightweight YOLO variants underscore the importance of task-specific structural designs—such as dilated aggregation, multi-scale attention, and lightweight feature fusion—in advancing efficient perception for autonomous-driving applications.

Recent advances in lightweight network design reflect a shift from hand-crafted compression techniques toward structural innovation. Liu et al. introduced EfficientViT [32], which replaces standard Multi-Head Self-Attention with CGA (Cascaded Group Attention) to eliminate inter-head redundancy. By assigning each head a distinct feature split and cascading their outputs, CGA increases model capacity without adding parameters and proportionally reduces the FLOPs of the Q–K–V projections, achieving a strong accuracy–throughput trade-off for detection tasks. Dynamic convolution has also gained traction: Wen et al. [33] proposed EMSPConv (Efficient Multi-Scale Partial Convolution), an adaptive kernel that learns position-specific weights across multiple scales and works with an efficient head design to slim the YOLOv8 detection stage while enhancing multi-scale feature representation. Inspired by PConv (Partial Convolution), Chen et al. [34] exploited feature redundancy by applying standard convolution only to a subset of channels while keeping the remaining channels unchanged; integrating this PConv-based unit into both the backbone and neck significantly reduces FLOPs. In addition, NAS (Neural Architecture Search) has increasingly been used to automatically generate lightweight and hardware-efficient topologies, further driving progress in lightweight model design and cross-domain generalization.

In summary, research—from classical techniques such as pruning, quantization, and knowledge distillation to recent advances in Transformers, dynamic convolutions, NAS, and lightweight head design—shows that model lightweighting has become a key development trend in object detection. While existing lightweight YOLO variants primarily rely on backbone substitution or shallow architectural scaling, the proposed C2f-Faster and EfficientHead modules address YOLO’s internal structural bottlenecks by reducing redundant channel computation in C2f and streamlining multi-scale prediction in the detection head. These refinements provide a more intrinsic and effective form of lightweight optimization, offering solid theoretical grounding and empirical evidence for high-performance, computationally efficient detection in autonomous-driving scenarios.

## 3. Methods

### 3.1. Overall Architecture of FE-YOLOv8

In object detection, models must achieve high accuracy while maintaining efficient inference. Although YOLOv8 performs strongly on small objects and in complex scenes, its C2f structure in the backbone and neck contains numerous skip connections and split operations, resulting in redundant computation and increased parameter cost. Moreover, its decoupled detection head—separating classification and regression branches—introduces additional computational overhead. These factors collectively constrain the inference efficiency of YOLOv8 and highlight the need for more lightweight architectural refinements. To mitigate these issues, lightweight strategies such as pruning, quantization, and knowledge distillation have been explored. While effective in reducing model size, these methods often compromise accuracy or require additional training, making them less suitable for high-precision detection tasks such as autonomous driving. As a result, generic lightweight techniques remain insufficient to simultaneously achieve high detection accuracy and strong inference efficiency.

**Figure 1:**
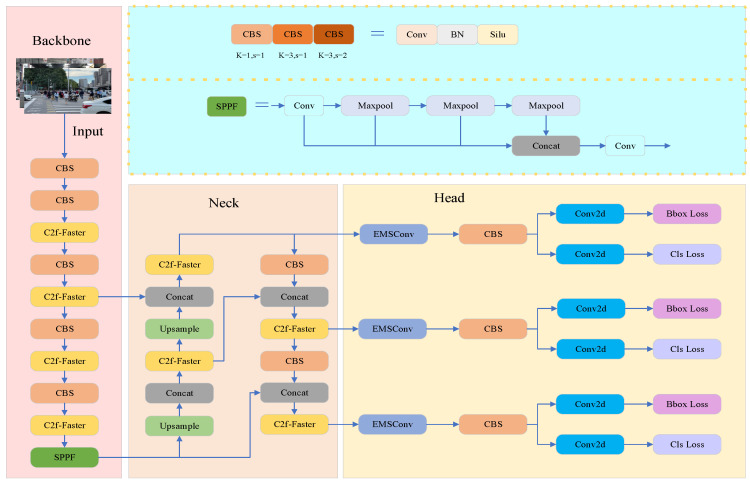
FE-YOLOv8 Model Architecture Diagram.

To address this limitation, we propose FE-YOLOv8, a lightweight object detection model optimized through structural enhancements. As illustrated in Figure 1, the model integrates the C2f-Faster module into both the backbone and neck to reduce redundant computation and improve feature extraction efficiency. The detection head adopts an EfficientHead design, which incorporates EMSConv to enhance multi-scale feature fusion while maintaining a compact architecture. FE-YOLOv8 follows the classical hierarchical Backbone–Neck–Head paradigm: the backbone extracts foundational features, the neck aggregates multi-scale representations, and the head performs object classification and bounding box regression. Building on this framework, the C2f-Faster module replaces the original YOLOv8 C2f block to reduce parameter size and computational overhead, whereas EfficientHead reconstructs the detection head and strengthens multi-scale feature modeling through EMSConv. The CBS (Conv–BatchNorm–SiLU) module, composed of convolution, batch normalization, and SiLU activation, further enhances feature expressiveness while maintaining computational efficiency. Overall, FE-YOLOv8 achieves structural lightweighting while preserving the end-to-end advantages of YOLOv8.

### 3.2. C2f-Faster Architecture

In YOLOv8’s network architecture, the C2f module is widely employed in both the backbone and neck to enhance feature extraction capabilities. However, this module consists of multiple convolutional blocks and cross-layer connections. While it facilitates improved gradient flow, it inevitably increases computational overhead and parameter count. This characteristic is particularly prominent in lightweight object detection tasks, where the high computational complexity and redundant operations limit the model’s deployment on edge devices and in real-time scenarios.

In lightweight model research, architectures such as MobileNet, ShuffleNet, and GhostNet commonly employ DWConv (depthwise separable convolutions) [35] or GConv (grouped convolutions) to reduce computational complexity. Although these operations effectively lower GFLOPs (Giga Floating-Point Operations), they often introduce additional memory-access overhead—arising from channel concatenation, feature refinement, and pooling procedures—which can substantially impact runtime efficiency. Therefore, controlling memory-access cost becomes as crucial as reducing computation when designing lightweight networks.

**Figure 2:**
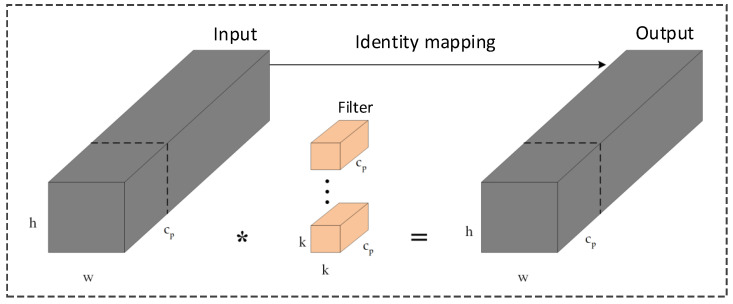
PConv Structure Diagram.

Chen et al. [34] introduced PConv, which leverages feature-map redundancy by applying standard convolution to only a subset of input channels while forwarding the remaining channels unchanged. This selective operation substantially reduces both computational cost and memory access. As illustrated in Figure 2, PConv preserves the same number of input and output channels but significantly decreases computational complexity. When the active-channel ratio p/c is set to 1/4, the computational cost drops to 1/16 of that of a full convolution, accompanied by a proportional reduction in memory-access demand. Based on this principle, the proposed C2f-Faster module replaces the full-channel Bottleneck units in YOLOv8’s original C2f block with Faster-Block units that use PConv as the spatial-mixing operator. In this design, only one quarter of the channels undergo a 3×3 spatial convolution, whereas the remaining channels follow an identity path and are fused afterward. Consequently, C2f-Faster maintains the kernel size, stride, and block depth of the original C2f structure while reducing the proportion of channels participating in spatial convolution from 100% to 25%. This preserves spatial resolution and structural compatibility with YOLOv8 while markedly decreasing FLOPs and memory-access cost.

**Figure 3:**
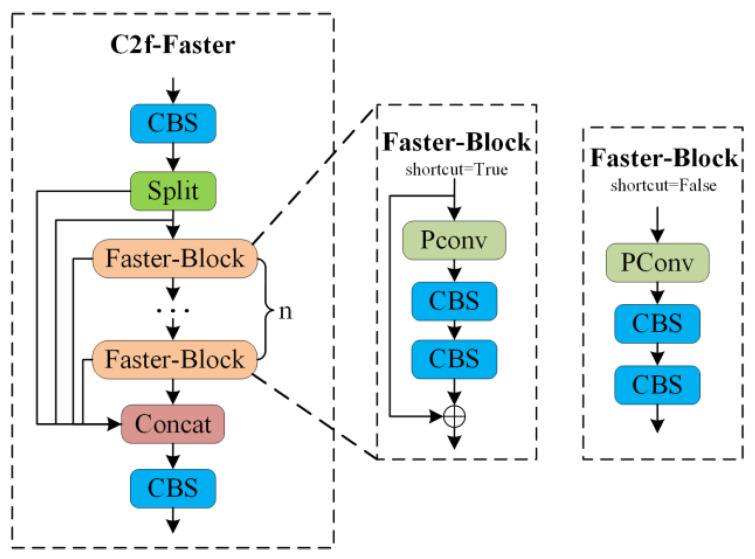
C2f-Faster Structure Diagram.

In the proposed C2f-Faster module, PConv is integrated with standard convolution to construct a Faster-Block that replaces the bottleneck components in the original YOLOv8 C2f modules (see Figure 3). This lightweight substitution applied to both the backbone and the neck reduces parameters and computational complexity while maintaining stable gradient propagation. Overall, the design achieves an effective balance between feature-extraction efficiency and model compactness.

### 3.3. Efficient Head Structure

**Figure 4:**
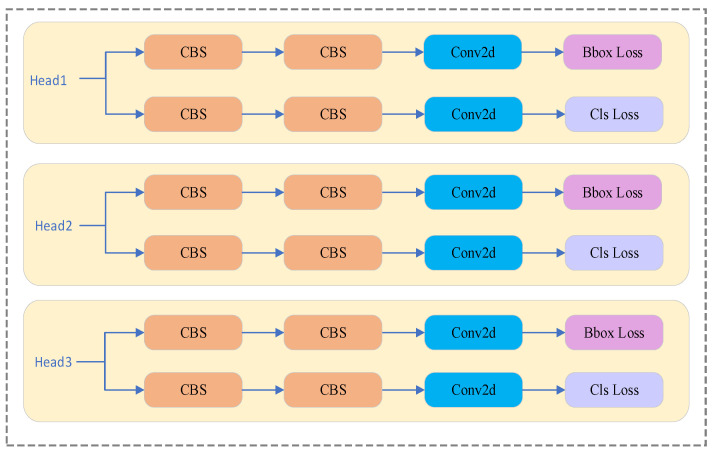
Head Structure Diagram of YOLOv8.

YOLOv8 adopts a decoupled head architecture in which the classification and regression branches are separated to mitigate representation conflict during feature learning. Compared with earlier coupled-head designs, this structure accelerates convergence and achieves a more balanced trade-off between classification performance and localization accuracy. In addition, YOLOv8 integrates DFL (Distributional Focal Loss) into the regression branch by expanding the output dimensionality to 4×reg_max, thereby improving the granularity and precision of bounding-box predictions. The overall architecture is illustrated in Figure 4.

Furthermore, YOLOv8 employs an anchor-free paradigm that eliminates the reliance on predefined bounding-box priors and thus improves adaptability to objects with diverse aspect ratios, scales, and deformation patterns. Nevertheless, this design may reduce recall in scenes characterized by dense object overlap or cluttered backgrounds, where anchor-based priors can provide useful spatial constraints. Although YOLOv8 is considered a lightweight model, the convolutional structure of its decoupled detection head still introduces noticeable computational cost, accounting for nearly 20% of the total parameters and FLOPs. Consequently, the detection head becomes a major performance bottleneck within the overall architecture.

To further reduce the computational cost of the detection head and improve multi-scale feature fusion efficiency, this paper proposes an EfficientHead architecture built upon YOLOv8. The key idea is to integrate EMSConv (Efficient Multi-Scale Convolution) into the head stem to reorganize and streamline the original convolutional pipeline. Specifically, the four parallel CBS (Convolution–Batch Normalization–SiLU) branches in the YOLOv8 head are replaced with a cascaded “EMSConv + CBS” structure, which substantially decreases redundant computation while retaining sufficient representational capacity for dense and small-object scenarios.

**Figure 5:**
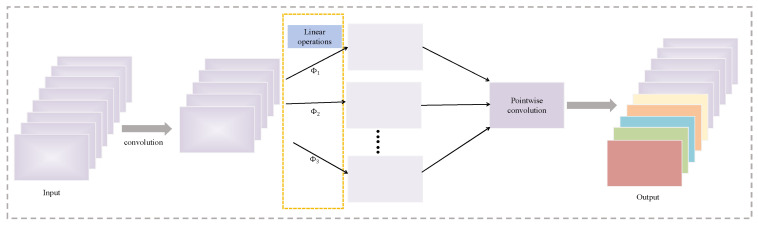
EMSConv Schematic Diagram.

In the proposed design, EMSConv is independently applied at each detection scale (P3, P4, and P5) as a lightweight spatial-enhancement operator preceding the classification and regression branches. To maintain architectural consistency, all scales adopt the same stem design; however, each scale-specific head retains its own parameters and processes its own feature map without altering YOLOv8’s original multi-scale prediction strategy. The overall structure of the EfficientHead is presented in Figure 5.

The design of EMSConv integrates key concepts from both GhostNet and MobileNet to exploit feature redundancy while improving cross-channel interaction efficiency. Specifically, GhostNet’s strategy is utilized to suppress redundant intermediate representations and retain only informative features, thereby reducing unnecessary computation. MobileNet’s pointwise convolution is incorporated to enhance channel-wise mixing. Furthermore, EMSConv employs multi-scale convolutional branches to better capture objects of varying sizes.

Within the EfficientHead, EMSConv functions as the primary spatial-mixing unit in the head stem. It first applies a standard convolution to the incoming feature map, after which the resulting tensor is divided into k subgroups. Each subgroup is processed by a lightweight linear transformation Φ, instantiated with convolution kernels of size 1×1, 3×3, 5×5, or 7×7, depending on its assigned branch. A final pointwise convolution aggregates these transformed subgroups and performs channel fusion, producing an output feature map enriched with hierarchical multi-scale information. This integration enables EfficientHead to strengthen multi-scale representation while maintaining a compact computational profile.

Complexity Analysis FLOPs (Floating-Point Operations): This metric quantifies the computational complexity of a model, where K denotes the kernel size of the convolution operation. The calculation formula is provided in Equation (1).

(1) $$\mathrm{FLOPs}=C_{out}\times H_{out}\times W_{out}\times C_{in}\times K\times K$$

- $C_{out}$: number of output channels,
- $C_{\mathrm{in}}$: number of input channels,
- $H_{\mathrm{out}}$: height of the output feature map,
- $W_{\mathrm{out}}$: width of the output feature map,
- K: kernel size of the convolution.

To examine the complexity reduction achieved by EMSConv, the FLOPs ratio between a standard convolution and EMSConv is expressed as

(2) $$r_{s}=\frac{c\cdot k\cdot k}{\frac{1}{s}\cdot c\cdot k\cdot k+\frac{s-1}{s}\cdot d\cdot d}\approx \frac{s\cdot c}{s+c-1}\approx s$$

- c: number of input channels,
- k: kernel size of the standard convolution,
- s: number of feature maps generated per input channel (expansion factor),
- d: kernel size of the linear operator Φ in EMSConv,
- $r_{s}$: theoretical FLOPs reduction ratio.

Assuming one identity mapping and s−1 EMSConv-generated feature maps, the numerator of Equation (2) represents the FLOPs of a standard convolution, while the denominator corresponds to the FLOPs of EMSConv. The first term in the denominator reflects the standard convolution applied to 1/s of the channels, and the second term accounts for the lightweight linear transformations applied to the remaining s−1 subgroups. Since the number of channels c is typically much larger than s, the denominator is dominated by c, making the reduction ratio $r_{s}$ approximately proportional to s. The term d×d denotes the kernel size of the linear transformation, which is generally comparable to the standard kernel size k×k. This shows that EMSConv substantially reduces computational complexity by replacing most spatial convolutions with lightweight linear operations.

The EfficientHead, built upon the EMSConv design, substantially reduces both the parameter count and computational complexity of the detection head while enhancing multi-scale feature fusion. Experimental results show that this design achieves faster inference with minimal loss in detection accuracy, thereby improving the overall efficiency and effectiveness of the model.

## 4. Experiment and Results

### 4.1. Hardware Platform and Parameters

All training and evaluation experiments were conducted on a workstation equipped with an Intel^®^ Xeon^®^ E5-2680 v4 CPU (14 physical cores), an NVIDIA GeForce RTX 3080 Ti GPU with 12 GB of GDDR6 memory (driver version 535.129.03), and 32 GB of DDR4 RAM. The software environment included Python 3.8, PyTorch 1.13.1, and CUDA 11.6 (see Table 1).

### 4.2. Dataset (Shown in Supplementary Materials) 

SODA-10M is a large-scale 2D autonomous driving dataset released in 2021 by Huawei Noah’s Ark Lab and Sun Yat-sen University. It provides annotations for Pedestrian, Cyclist, Car, Truck, and Tram, and includes diverse road types, weather conditions, and time periods [36]. This diversity makes SODA-10M suitable for pretraining and for use as additional data in semi-supervised learning for downstream perception tasks.

The BDD100K dataset is a publicly available driving-scene dataset released in 2018 by the AI Lab at the University of California, Berkeley. It covers a broad range of real-world driving scenarios and is widely used in road object detection research [37]. In this study, 1000 images were selected from BDD100K to evaluate the generalization performance of the proposed model.

### 4.3. Experimental Validation and Results Analysis

#### 4.3.1. Model Validation Analysis

To validate the effectiveness of the proposed lightweight improvements, this section adopts YOLOv8 as the baseline and conducts both ablation and comparative experiments. The C2f modules in the backbone and neck are enhanced by integrating PConv within the bottleneck architecture, effectively reducing parameter count and computational complexity. Concurrently, EMSConv are incorporated into the detection head, which is subsequently redesigned to improve multi-scale feature fusion and further enhance the model’s lightweight performance.

The training settings are summarized as follows. SGD (Stochastic Gradient Descent) was used as the optimizer, with an initial learning rate of 0.01 and a weight decay of 0.0005. A warm-up phase was applied at the beginning of training, followed by a scheduled learning-rate decay. The batch size was set to 16, and all input images were resized to 640 × 640. Data loading employed 8 workers. Mosaic and MixUp were used for data augmentation, with Mosaic disabled during the final 20 epochs to stabilize convergence. AMP (Automatic Mixed Precision) was enabled throughout training. The model was trained for 100 epochs. During testing, the confidence threshold and IoU threshold were set to 0.25 and 0.70, respectively (see Table 2).

**Figure 6:**
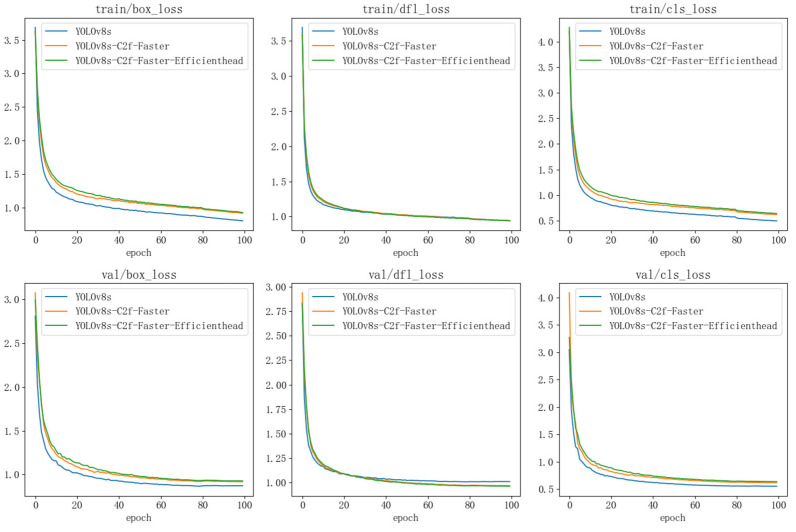
Training and validation loss curves for the lightweight YOLOv8 model.

Figure 6 presents the training loss curves for the different models. Both proposed lightweight variants exhibit a smooth and consistent decrease in loss, ultimately reaching stable convergence. This demonstrates the robustness of the training process and confirms the effectiveness of the structural optimizations, providing a reliable basis for the subsequent comparison of inference speed and detection accuracy.

#### 4.3.2. Ablation Experiment Results

**Figure 7:**
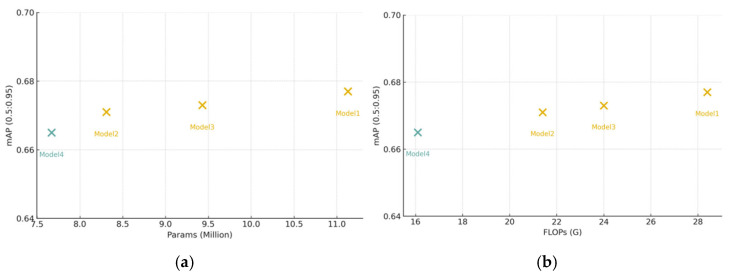
Scatter plots of model complexity and accuracy: (**a**) Params–mAP; (**b**) FLOPs–mAP.

Table 3 presents an ablation study isolating the effects of C2f-Faster, EfficientHead, and their combination. Integrating C2f-Faster into the backbone (Model 2) reduces parameters and FLOPs by 25.34% and 24.65%, respectively, with only marginal changes in Precision and mAP, demonstrating that the proposed lightweight backbone preserves detection capability. Applying EfficientHead alone (Model 3) yields a similar trend, reducing model size by approximately 15% and maintaining accuracy within a 0.01 range of the baseline. Combining both components (Model 4) achieves the most substantial compression—31.09% fewer parameters and 43.31% lower FLOPs—while incurring only a ~1-point drop in mAP. Figure 7 corroborate these results, showing that Model 4 lies closest to the optimal efficiency–accuracy frontier.

Figure 7 illustrate the Params–mAP and FLOPs–mAP distributions of the evaluated models. Model 1 achieves slightly higher mAP but incurs the greatest parameter and computational overhead. Models 2 and 3 achieve mAP values comparable to Model 1 while substantially reducing model size and complexity. Model 4 attains the lowest parameter count and FLOPs, with a mAP only ~1 percentage point lower than the other variants—an insignificant difference in practical scenarios. Consequently, Model 4 achieves the highest degree of lightweighting and inference acceleration with negligible accuracy loss, offering an advantageous precision–complexity trade-off.

Overall, the quantitative results in Table 3 and the visual patterns in Figure 7 demonstrate that the proposed C2f-Faster module and EfficientHead significantly improve the lightweight performance of the baseline YOLOv8 architecture. Notably, Model 4 provides the most substantial reductions in both parameters and FLOPs while preserving detection accuracy.

#### 4.3.3. Comparative Experiments Results

To further validate the effectiveness of the proposed FE-YOLOv8, we conducted a comparative evaluation against four state-of-the-art lightweight paradigms reported in the recent literature. Table 4 shows that FE-YOLOv8 contains 7.67 M parameters and 16.1 G FLOPs. Although its parameter count is slightly higher than those of the ShuffleNetV2- and MobileNetV3-based variants, it requires significantly fewer floating-point operations. At the same time, FE-YOLOv8 achieves markedly superior detection performance across all evaluation metrics. These results indicate that the proposed architecture attains a more favorable balance between computational efficiency and predictive accuracy, reinforcing its advantages within the lightweight model design space.

**Figure 8:**
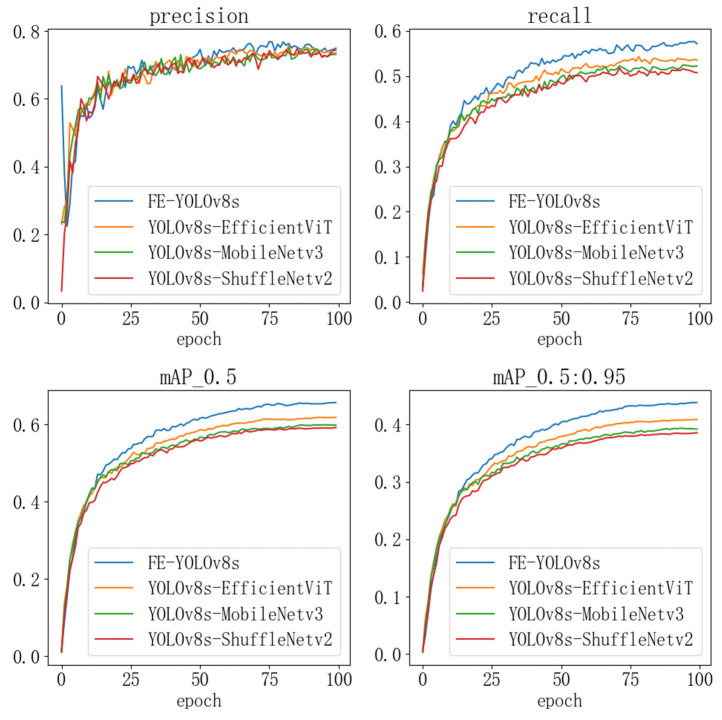
Performance Comparison of Lightweight Experimental Models.

As shown in Figure 8, In the figure, the blue curve represents FE-YOLOv8; the yellow curve corresponds to the EfficientViT-based variant; the green curve denotes the MobileNetV3-based architecture; and the red curve illustrates the ShuffleNetV2-based configuration. FE-YOLOv8 consistently outperforms the three competing lightweight paradigms across all monitored metrics—Precision, Recall, mAP@0.5, and mAP@0.5:0.95—throughout the entire training process. The model achieves both faster convergence and higher final accuracy, demonstrating that the proposed architectural refinements not only improve detection performance but also enhance the stability of the training process.

Taken together, the results in Table 3 and Figure 8 demonstrate that FE-YOLOv8 surpasses existing lightweight approaches in terms of parameter count, computational complexity, and detection accuracy. These findings confirm the effectiveness of the proposed framework and highlight its strong potential for lightweight object-detection applications.

To further validate the performance of the proposed lightweight detector FE-YOLOv8, a comparative study was conducted under unified experimental settings and hyperparameter configurations. The evaluation pool comprises eight representative architectures—SSD, Faster R-CNN, CenterNet, YOLOv5s, YOLOv6s, YOLOv7s, YOLOv8s, YOLOv9s, and YOLOv11s—enabling a comprehensive comparison in terms of parameter count, FLOPs, and mean Average Precision (mAP). To ensure fairness and reproducibility, all reported metrics correspond to the best performance across multiple training runs, with identical data augmentation, optimizer settings, and training schedules applied to all models.

As summarised in Table 5, FE-YOLOv8 achieves competitive or slightly higher detection accuracy than most baselines while maintaining relatively low computational complexity. Notably, it presents a more favorable accuracy–efficiency trade-off than the original YOLOv8s under the same input resolution and training protocol. These results demonstrate that FE-YOLOv8 is a strong lightweight alternative within the YOLO family and further confirm the effectiveness of the proposed architectural refinements.

#### 4.3.4. Analysis of Model Lightweighting Detection Performance

**Figure 9:**
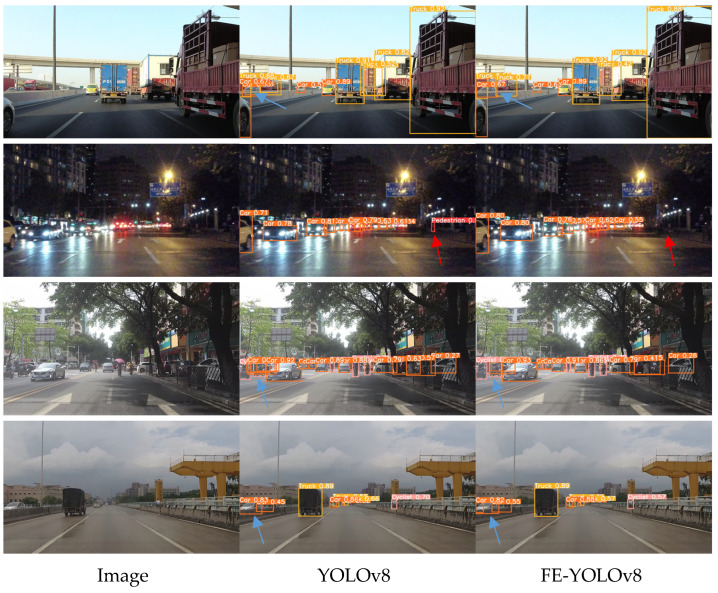
Visualization of detection results produced by the proposed FE-YOLOv8 model.

To intuitively evaluate the practical performance of the improved FE-YOLOv8 model, four representative scenarios—sunny, nighttime, urban street scenes, and overcast—were selected for visualization. As shown in Figure 9, the first column presents the ground-truth annotations, while the second and third columns show the detection results of YOLOv8 and FE-YOLOv8, respectively. In the visualizations, blue arrows denote false positives, whereas red arrows indicate missed detections.

In the sunny scenario, YOLOv8 produces multiple false detections of vehicles, whereas FE-YOLOv8 correctly identifies the objects without generating redundant bounding boxes. Under nighttime conditions, YOLOv8 successfully detects pedestrians that FE-YOLOv8 fails to recognize. In the urban street-scene scenario, YOLOv8 yields several false positives, all of which are eliminated by the improved FE-YOLOv8 model. In the overcast scenario, YOLOv8 accurately captures the corresponding objects, while FE-YOLOv8 produces several inaccurate bounding boxes. These qualitative results indicate that FE-YOLOv8 achieves detection performance generally comparable to that of the original YOLOv8, with each model exhibiting advantages and limitations across different illumination, weather, and scene conditions.

#### 4.3.5. Evaluation of Model Generalization and Visual Results

**Figure 10:**
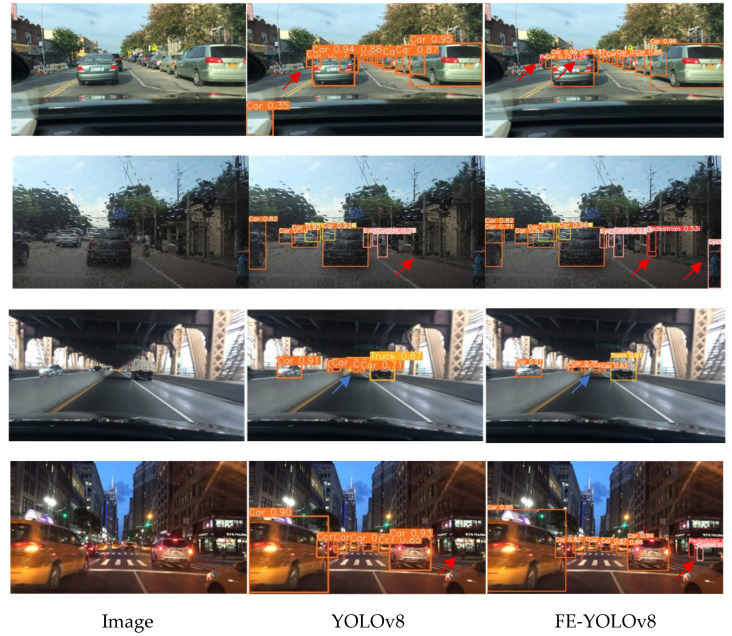
Visualized generalization detection results of the proposed FE-YOLOv8 model.

To evaluate the generalization capability of the proposed model under visually challenging real-world conditions, four representative scenarios from the BDD100K dataset—sunny, rainy, elevated-bridge, and dusk—were selected for qualitative comparison. As illustrated in Figure 10, each scenario is presented in three columns: the raw image, YOLOv8 predictions, and FE-YOLOv8 outputs.

In the visualizations, red arrows indicate missed detections and blue arrows indicate false detections produced. In the sunny scene, YOLOv8 misses several vehicles marked by the red arrows, whereas FE-YOLOv8 successfully detects them with accurate and compact bounding boxes. In the rainy scene, YOLOv8 again exhibits missed detections, while FE-YOLOv8 correctly identifies the pedestrians. In the elevated-bridge scenario, YOLOv8 produces false detections, as highlighted by the blue arrows, whereas the improved FE-YOLOv8 eliminates these errors. In the dusk scene, YOLOv8 shows missed detections under low illumination, whereas FE-YOLOv8 correctly captures the corresponding objects. These qualitative results demonstrate that FE-YOLOv8 provides stronger cross-condition robustness and improved generalization, particularly under variations in illumination, weather, and scene geometry.

In terms of computational efficiency, FE-YOLOv8 records average latencies of 0.3 ms for pre-processing, 1.5 ms for inference, and 1.3 ms for post-processing, compared with 0.3 ms, 2.0 ms, and 1.7 ms for YOLOv8 under identical hardware and software settings. These results show that FE-YOLOv8 maintains strong detection accuracy while reducing inference and post-processing overhead.

## 5. Summary

The proposed FE-YOLOv8 architecture achieves a principled lightweight redesign of YOLOv8 by integrating the C2f-Faster module into both the backbone and neck and replacing the original detection head with the more parameter-efficient EfficientHead. Extensive experiments show that these modifications reduce the parameter count by 31.09% and the FLOPs by 43.31% while maintaining baseline-level detection accuracy, demonstrating an improved accuracy–compactness trade-off. Benchmark comparisons further indicate that FE-YOLOv8 outperforms widely used lightweight backbones—including MobileNet, ShuffleNet, and EfficientViT—in both mAP and inference throughput. Qualitative evaluations under varying illumination and visually cluttered conditions additionally confirm the model’s robustness and strong cross-condition generalization.

## Figures and Tables

**Table 1:** Experimental Environment Requirements.

Name	Parameter Specifications
CPU	Intel^®^ Xeon^®^ CPU E5-2680v4
GPU	3080 Ti-12G
Memory	32 GB
CUDA version	11.6.0
Python	3.8
Pytorch	1.13.1

**Table 2:** Training Configuration Requirements.

Item	Setting
Optimizer	SGD
Initial learning rate	0.01
Learning rate schedule	Warm-up at the beginning followed by a decay schedule during training
Weight decay	0.0005
Batch size	16
Input image size	640 × 640
Data loading workers	8 threads
Data augmentation	Mosaic + MixUp
Mosaic scheduling	Mosaic disabled in the final 20 epochs
Precision	Automatic Mixed Precision (AMP)
Training epochs	100
Confidence threshold (test)	0.25
IoU threshold (test)	0.70

**Table 3:** Performance Comparison of the Lightweight YOLOv8 Model.

Model	C2f-Faster	EfficientHead	P	R	mAP_0.5	Param	FLOPs
Model 1			0.758	0.601	0.677	11.13 M	28.4 G
Model 2	√		0.769	0.581	0.671	8.31 M	21.4 G
Model 3		√	0.765	0.595	0.673	9.43 M	24.0 G
Model 4	√	√	0.768	0.588	0.665	7.67 M	16.1 G

**Table 4:** Comparison of Lightweight Models.

Model	Param	FLOPs
YOLOv8-ShuffleNetv2	6.39 M	16.5 G
YOLOv8-MobileNetv3	6.74 M	16.4 G
YOLOv8-EfficientViT	8.39 M	20.4 G
FE-YOLOv8	**7.67 M**	**16.1 G**

**Table 5:** Experimental comparison of different network model.

Model	P	R	mAP_0.5	mAP_0.5: 0.95	Param	FLOPs
SSD	0.693	0.515	0.608	0.345	4.58 M	10.2 G
Faster-RCNN	0.742	0.560	0.650	0.380	46.3 M	138.7 G
CenterNet	0.725	0.550	0.640	0.365	32.1 M	40.2 G
YOLOv5s	0.742	0.560	0.650	0.375	7.2 M	15.0 G
YOLOv6s	0.757	0.573	0.664	0.388	11.2 M	23.7 G
YOLOv7s	0.765	0.580	0.670	0.442	19.8 M	45.5 G
YOLOV8s	0.758	0.601	0.677	0.445	11.13 M	28.4 G
YOLOV9s	0.766	0.592	0.665	0.440	8.4 M	27.6 G
YOLOV11s	0.761	0.610	0.668	0.439	10.6 M	21.4 G
FE-YOLOv8	0.768	0.588	0.665	0.438	7.67 M	16.1 G

## Data Availability

Data are contained within the article.

## Supplementary Materials

The public datasets referenced in Section 4.2 Dataset may be downloaded from the following URLs: http://bdd-data.berkeley.edu (accessed on 1 June 2024) and https://soda-2d.github.io/index.html (accessed on 1 October 2024).

## References

[1] Yurtsever E., Lambert J., Carballo A., Takeda K. (2020). A Survey of Autonomous Driving: Common Practices and Emerging Technologies. IEEE Access.

[2] Chi X., Sun Y., Zhao Y., Lu D., Gao Y., Zhang Y. (2024). An Improved YOLOv8 Network for Detecting Electric Pylons Based on Optical Satellite Image. Sensors.

[3] Bachute M.R., Subhedar J.M. (2021). Autonomous Driving Systems: A Systematic Literature Review. J. King Saud Univ.—Comput. Inf. Sci..

[4] Szegedy C., Liu W., Jia Y., Sermanet P., Reed S., Anguelov D., Erhan D., Vanhoucke V., Rabinovich A., Liu W. Going deeper with convolutions. Proceedings of the IEEE Conference on Computer Vision and Pattern Recognition.

[5] Jocher G., Stoken A., Borovec J., Changyu L., Hogan A., NanoCode012, ChristopherSTAN, Laughing, tkianai, lorenzomammana (2020). Ultralytics YOLOv5: Real-Time Object Detection.

[6] Wang C.-Y., Bochkovskiy A., Liao H.-Y.M. YOLOv7: Trainable Bag-of-Freebies Sets New State-of-the-Art for Real-Time Object Detectors. Proceedings of the IEEE/CVF Conference on Computer Vision and Pattern Recognition (CVPR).

[7] Yaseen M. (2024). What Is YOLOv8: An In-Depth Exploration of YOLOv8 for Object Detection. arXiv.

[8] Khanam R., Hussain M. (2024). YOLOv11: An Overview of the Key Architectural Enhancements. arXiv.

[9] Lyu Z., Bai H., Zhong Z., Jia X., Cao J. (2024). A Survey of Model Compression Strategies for Object Detection. Multimed. Tools Appl..

[10] Cheng Y., Wang D., Zhou P., Zhang T. (2018). A Survey of Model Compression and Acceleration for Deep Neural Networks. IEEE Signal Process. Mag..

[11] Jacob B., Kligys S., Chen B., Zhu M., Tang M., Howard A., Adam H., Kalenichenko D. Quantization and Training of Neural Networks for Efficient Integer-Arithmetic-Only Inference. Proceedings of the IEEE/CVF Conference on Computer Vision and Pattern Recognition (CVPR).

[12] Boutros F., Damer N., Kuijper A. Quantface: Towards lightweight face recognition by synthetic data low-bit quantization. Proceedings of the 26th International Conference on Pattern Recognition (ICPR).

[13] Park J., Qian X., Jo Y., Sung W. Low-latency lightweight streaming speech recognition with 8-bit quantized simple gated convolutional neural networks. Proceedings of the ICASSP 2020 IEEE International Conference on Acoustics, Speech and Signal Processing (ICASSP).

[14] Peng D., Wang T. (2019). Pruning algorithm based on GoogLeNet model. Control Decis..

[15] Xu J. (2019). Research on neural network compression technology based on model pruning. Inf. Commun..

[16] Xiang H., Yu S., Li P., Li W., Wu E., Sheng B. SlimFluid-Net: Fast fluid simulation using ADMM pruning. Proceedings of the Computer Graphics International Conference.

[17] Blakeney C., Li X., Yan Y., Zong Z. (2020). Parallel blockwise knowledge distillation for deep neural network compression. IEEE Trans. Parallel Distrib. Syst..

[18] Kang M., Kang S. (2021). Data-free knowledge distillation in neural networks for regression. Expert Syst. Appl..

[19] Tung F., Mori G. Similarity-preserving knowledge distillation. Proceedings of the IEEE/CVF International Conference on Computer Vision.

[20] Howard A.G., Zhu M., Chen B., Kalenichenko D., Wang W., Weyand T., Andreetto M., Adam H. (2017). Mobilenets: Efficient convolutional neural networks for mobile vision applications. arXiv.

[21] Sandler M., Howard A., Zhu M., Zhmoginov A., Chen L. MobileNetV2: Inverted residuals and linear bottlenecks. Proceedings of the IEEE Conference on Computer Vision and Pattern Recognition.

[22] Howard A., Sandler M., Chen B., Wang W., Chen L.-C., Tan M., Chu G., Vasudevan V., Zhu Y., Pang R. Searching for MobileNetV3. Proceedings of the IEEE/CVF International Conference on Computer Vision (ICCV).

[23] Zhang X., Zhou X., Lin M., Sun J. ShuffleNet: An extremely efficient convolutional neural network for mobile devices. Proceedings of the IEEE Conference on Computer Vision and Pattern Recognition (CVPR).

[24] Ma N., Zhang X., Zheng H.-T., Sun J. ShuffleNet V2: Practical guidelines for efficient CNN architecture design. Proceedings of the European Conference on Computer Vision (ECCV).

[25] Han K., Wang Y., Tian Q., Guo J., Xu C., Xu C. GhostNet: More features from cheap operations. Proceedings of the IEEE/CVF Conference on Computer Vision and Pattern Recognition (CVPR).

[26] He Q., Xu A., Ye Z., Zhou W., Cai T. (2023). Lightweight YOLOX for Autonomous Driving. Sensors.

[27] Shi P., Li L., Qi H., Yang A. (2023). MobileNetV2_CA: A Lightweight Object Detection Network in Autonomous Driving. Technologies.

[28] Yang M., Fan X. (2024). YOLOv8-Lite: A Lightweight Object Detection Model for Real-Time Autonomous Driving Systems. J. Real-Time Image Process..

[29] Cui S., Liu F., Wang Z., Zhou X., Yang B., Li H., Yang J. (2024). DAN-YOLO: A Lightweight and Accurate Object Detector Using Dilated Aggregation Network for Autonomous Driving. Electronics.

[30] Li M., Liu X., Chen S., Yang L., Du Q., Han Z., Wang J. (2024). MST-YOLO: Small Object Detection Model for Autonomous Driving. Sensors.

[31] Kalgaonkar P., El-Sharkawy M. (2023). An Improved Lightweight Network Using Attentive Feature Aggregation for Object Detection in Autonomous Driving. J. Low Power Electron. Appl..

[32] Liu X., Peng H., Zheng N., Yang Y., Hu H., Yuan Y. EfficientViT: Memory efficient vision transformer with cascaded group attention. Proceedings of the IEEE/CVF Conference on Computer Vision and Pattern Recognition (CVPR).

[33] Wen G., Li M., Luo Y., Shi C., Tan Y. (2024). The improved YOLOv8 algorithm based on EMSPConv and SPE-head modules. Multimed. Tools Appl..

[34] Chen J., Kao S.-H., He H., Zhuo W., Wen S., Lee C.-H., Chan S.-H.G. Run, don’t walk: Chasing higher FLOPS for faster neural networks. Proceedings of the IEEE/CVF Conference on Computer Vision and Pattern Recognition (CVPR).

[35] Chollet F. Xception: Deep Learning with Depthwise Separable Convolutions. Proceedings of the IEEE Conference on Computer Vision and Pattern Recognition (CVPR).

[36] Han J., Liang X., Xu H., Chen K., Hong L., Mao J., Ye C., Zhang W., Li Z., Liang X. (2021). SODA10M: A Large-Scale 2D Self/Semi-Supervised Object Detection Dataset for Autonomous Driving. arXiv.

[37] Yu F., Chen H., Wang X., Xian W., Chen Y., Liu F., Madhavan V., Darrell T. (2020). BDD100K: A Diverse Driving Dataset for Heterogeneous Multitask Learning. arXiv.

